# Towards comprehensive structural motif mining for better fold annotation in the "twilight zone" of sequence dissimilarity

**DOI:** 10.1186/1471-2105-10-S1-S46

**Published:** 2009-01-30

**Authors:** Yi Jia, Jun Huan, Vincent Buhr, Jintao Zhang, Leonidas N Carayannopoulos

**Affiliations:** 1 Department of Electrical Engineering & Computer Science, University of Kansas, Lawrence, KS, 66045, USA; 2 Department of Molecular Biosciences, The University of Kansas, Lawrence, KS 66046, USA; 3 School of Medicine, Washington University in St. Louis, St. Louis, MO 63130, USA

## Abstract

**Background:**

Automatic identification of structure fingerprints from a group of diverse protein structures is challenging, especially for proteins whose divergent amino acid sequences may fall into the "twilight-" or "midnight-" zones where pair-wise sequence identities to known sequences fall below 25% and sequence-based functional annotations often fail.

**Results:**

Here we report a novel graph database mining method and demonstrate its application to protein structure pattern identification and structure classification. The biologic motivation of our study is to recognize common structure patterns in "immunoevasins", proteins mediating virus evasion of host immune defense. Our experimental study, using both viral and non-viral proteins, demonstrates the efficiency and efficacy of the proposed method.

**Conclusion:**

We present a theoretic framework, offer a practical software implementation for incorporating prior domain knowledge, such as substitution matrices as studied here, and devise an efficient algorithm to identify approximate matched frequent subgraphs. By doing so, we significantly expanded the analytical power of sophisticated data mining algorithms in dealing with large volume of complicated and noisy protein structure data. And without loss of generality, choice of appropriate compatibility matrices allows our method to be easily employed in domains where subgraph labels have some uncertainty.

## Background

Genomics efforts continue to yield a myriad of new protein sequences. Among the most valuable are those expressed by mammalian pathogens, organisms that successfully grow and disseminate despite a hostile host immunologic environment. A subset of pathogen-encoded proteins, "immunoevasins", facilitate this success by mediating cellular adhesion and entry, and by distorting the interactions of host receptors and cell-surface ligands [1]. Study of immunoevasins gives insight into host-defense mechanisms, insight that can help guide development of therapies and vaccines against refractory organisms [2].

Though immunoevasins frequently possess protein-recognition domain (PRD) folds common to mammalian proteins of immunologic importance, their divergent amino acid sequences may fall into the "twilight-" or "midnight-" zones where pair-wise sequence identities to known sequences fall below 25% and purely sequence-based attempts at annotations often fail [3,4].

To better annotate these, and any other highly divergent sequences, more generally, some means of explicitly incorporating three-dimensional structural information into the sequence evaluation is required. Inclusion of even rudimentary structural considerations enhances the performance of sequence scoring heuristics such as local alignment tools [5] and hidden Markov models (HMM) [6]. Indeed an HMM constrained with crystallographically determined secondary structure data allowed discovery of a previously unsuspected MHC class I-like immunoevasin in the genomes of orthopoxviruses [7]. A vast literature covers various schemes for structural data incorporation and fold classification. Nevertheless, much progress remains to be made [8].

We are pursuing an approach whereby structural patterns common to a protein fold are collected, assessed for their classification value, and mapped onto statistical models of protein sequences (e.g. HMMs, support vector machines (SVMs), and conditional random fields). As a first step, a comprehensive and objective means is required of identifying and assessing the above common structure patterns, or structure fingerprints.

Automatic identification of structure fingerprints from a group of diverse protein structures is challenging for a number of reasons. First, we have only limited knowledge about the possible location, composition, and geometric shape of these structure patterns. Second, protein structures are large geometric objects that typically contain hundreds of amino acids with thousands of atoms and chemical bonds. Third, due to accumulated mutations in evolution the same structure pattern may appear slightly different in different proteins. If we use terms from computer algorithm design, we say that the problem of automatic structure pattern identification is challenging since (1) the problem has a large combinatory search space (meaning patterns may occur in any part of a protein and in any subset of a group of proteins) and (2) we should use approximate matching rather than exact matching in retrieving such patterns (meaning that we should tolerate certain level of geometric distortion and amino acid mismatch in search for common structure patterns).

In this paper we demonstrate a novel data mining technique that efficiently extracts and scores structure pattern from diverse proteins. Specifically in our method, we encode a protein structure as a geometric graph where a node represents an amino acid residue and an edge represents a physical or a chemical interaction between a pair of residues. We encode structural motifs as subgraphs of a geometric graph and we identify conserved structure fingerprints by searching for frequently occurring approximately subgraphs in a group of graph represented proteins.

Our contributions in designing a new graph data mining method are to develop a solid theoretic framework, to offer a practical software implementation for incorporating prior domain knowledge, such as substitution matrices as studied here, and to devise an efficient algorithm to identify approximate matched frequent subgraphs. By doing so, we expanded the analytical power of data mining algorithms in dealing with large volume of complicated and noisy protein structure data. As evaluated in our driving biological application of recognizing common structure patterns in immunoevasins, our proposed method identifies many structure patterns and affords better structure classification accuracy compared to existing graph mining algorithms.

The rest of the paper is organized in the following way. In the *Related Work *section, we give an overview of related work on subgraph mining and protein structure pattern identification. In the *Methods *section, we introduce the technique about how to translate protein structures into graphs, provide our model for approximate subgraph mining, and present the details of our algorithm. In the *Results *section, we show an empirical study of the proposed algorithm using protein structure data sets. In the *Discussion *section, we discuss the biological significance of the structural motifs mined by our method. Finally in the *Conclusions *section, we conclude with a short discussion of our approach.

## Related work

There is an extensive body of literature on comparing and classifying proteins using multiple sequence or structure alignment, such as VAST [9] and DALI [10]. Here we focus on the recent algorithmic techniques for discovering structure motifs from protein structures. The methods can be classified into the following five types:

• Depth-first search, starting from simple geometric patterns such as triangles, progressively finding larger patterns [11-13].

• Geometric hashing, originally developed in computer vision, applied pairwise between protein structures to identify structure motifs [14-16].

• String pattern matching methods that encode the local structure and sequence information of a protein as a string, and apply string search algorithms to derive motifs [17-19].

• Delaunay Tessellation (DT) [20-22] partitioning the structure into an aggregate of non-overlapping, irregular tetrahedra thus identifying all unique nearest neighbor residue quadruplets for any protein [22].

• Graph matching methods comparing protein structures modeled as graphs and discovering structure motifs by finding recurring subgraphs [23-29].

Graph database mining is an active research field in data mining research. The goal of graph database mining is to locate useful and interpretable patterns in a large volume of graph data. Recent exact matching graph mining algorithms can be roughly divided into three categories. The first category uses the level-wise search strategy, which includes AGM [30] and FSG [31]. And the second category takes the depth-first search strategy, which includes gSpan [32] and FFSM [33]. The third category works by mining frequent trees, for which SPIN [34] and GASTON [35] are the representative. There are many other existing graph mining algorithms, and we refer to [36] for a recent survey.

Frequent subgraph mining with approximate matching capability has also been investigated. The current approximate subgraph mining algorithms can be divided into four categories: (1) proximity measures between graphs [37-39], (2) given a proximity measurement, compute representative frequent subgraphs [40], (3) pattern discovery in a single large graph [41], and (4) pattern discovery from a group of graphs. The last category is what we concentrate on. For algorithms in (4), SUBDUE [42] does not claim completeness. Monkey [43] handles only edge missing and edge label mismatch. Partially Labeled Graphs [44] uses a wild card method to handle node label mismatches. The algorithm may be viewed as a special case of our algorithm.

Different from the existing work, to our best knowledge, we are the first group that incorporates a probability matrix in a graph mining method. We also developed a general framework to fully utilize a probability matrix for approximate match, which we can apply to a number of different applications. In addition, we have developed two ways to demonstrate the statistical significance of the patterns mined from a graph database. Statistical significance is an important but often overlooked issue in evaluating the quality of identified pattern in frequent pattern mining. Finally we offered a practical implementation and evaluated its performance using the synthetic sets.

## Methods

In this section, we first briefly describe the technique that translates protein structures into graphs. Then we demonstrate our method called **APGM**(**AP**proximate **G**raph **M**ining) with two steps: introducing the theoretic model, and showing our algorithm in detail.

### Almost-Delaunay graph

**Figure 1:**
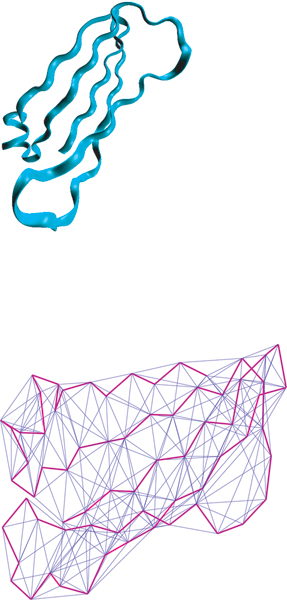
**3D structure and corresponding graph of one sample protein**. **Upper: **One segment of the 3D structure of the 1FP5A Immunoglobulin C1-type protein (the paired Fc*ε *3 and 4 domains of IgE). **Lower: **The corresponding graph. Vertices are *C*_*α *_atoms. Covalent edges are represented in heavy magenta while non-covalent edges defined by Almost Delaunay Tesselation(*ε *= 0.1) appear in thin blue.

Since the protein backbone trace defines the overall protein conformation, we choose the *C*_*α *_atoms as the nodes of protein graphs. Based on this simplified protein model, we compute edges using Almost-Delaunay Tesselation [45]. The Almost-Delaunay edges are a superset of the Delaunay edges. All nearest neighbor residues connected by Delaunay edges are defined using Delaunay Tessellation [46]. This tessellation is defined for a finite set of points by an empty sphere property: A pair of points is joined by an edge iff one can find an empty sphere whose boundary contains those two points. The definition of the Delaunay Tessellation depends on the precise coordinate values given to its points, but these coordinate values are not exact in the case of proteins due to measurement imprecision and atomic motions. In order to address this problem, Almost-Delaunay Edges are defined by relaxing the empty sphere property to say that a pair of points *p *and *q *is joined by an Almost-Delaunay edge with parameter *ε*, or AD(*ε*), if by perturbing all points by at most *ε*, *p *and *q *can be made to lie on an empty sphere. In Figure 1, we show one segment of the 3D structure and the corresponding AD graph of 1FP5A Immunoglobulin C1-type protein as an example. More detailed information is available in [45] and [47].

### Theoretic framework

#### Definition 1

*A ****labeled graph ****G is a 5-tuple G *= {*V*, *E*, Σ_*V*_, Σ_*E*_, *λ*) *where V is the set of vertices of G and E *⊆ *V *× *V is the set of undirected edges of G*. Σ_*V *_*and *Σ_*E*_* are (disjoint) sets of labels. And labeling function λ*: *V *→ Σ_*V *_∪ *E *→ Σ_*E *_*maps vertices and edges in G to their labels. A ****graph database ****D is a set of graphs*.

We also use *V*[*G*] to denote the node set of a graph *G *and *E*[*G*] to denote the edge set of *G*. We also use Σ_*V*[*G*] _to denote the node labels, Σ_*E*[*G*] _to denote edge labels, and *λ*_*G *_to denote the labeling function for a graph *G*. Before we introduce approximate matching, we define compatibility matrix, which offers a probability framework for approximate subgraph mining.

#### Definition 2

*A ****compatibility matrix ****M *= (*m*_*i*,*j*_) *is an n *× *n matrix indexed by symbols from a label set *Σ *(n *= |Σ|*). An entry m*_*i*,*j*_*(*0 ≤ *m*_*i*,*j *_≤ 1, Σ_*j*_*m*_*i*,*j *_= 1*) in M is the probability that the label i is replaced by the label j*.

A compatibility matrix *M *is *stable *if the diagonal entry is the largest one in the row (i.e. *M*_*i*,*i *_> *M*_*i*,*j*_, for all *j *≠ *i*). A compatibility matrix being stable means that any label *i *is more likely to be replaced by itself rather than by any other symbol. For our biological application, we consider substitution matrices as being, in essence, stable matrices since most or all rows fit the criterion. For example, in the BLOSUM62 substitution matrix, there is only one violation of the criterion – the row for methionine(MET). Hence for the rest of the discussion, we will treat substitution matrices as stable compatibility matrices.

**Figure 2:**
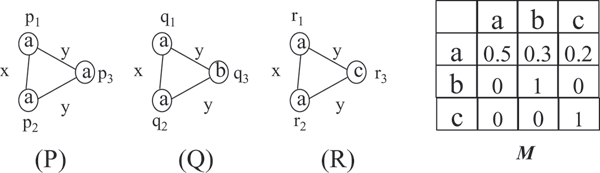
**Graph database and compatibility matrix**. Example of a graph database *D *and a compatibility matrix *M*.

**Example 1. ***We show a graph database D with three labeled graphs P, Q, R on the left side of *Figure 2. *In this database, the node label set is *{*a*, *b*, *c*} *and the edge label set is *{*x*, *y*}. *On the right part of *Figure 2, *we show a compatibility matrix M, which is a 2D matrix indexed by the set of node labels in D. The probability that the vertex label a is substituted by b is m*_*a*,*b *_= 0.3. *In M, we use probability *0 *to simplify the matrix. In reality these probabilities are never *0.

#### Definition 3

*A labeled graph G *= {*V*, *E*, Σ_*V*_, Σ_*E*_, *λ*} *is ****approximately subgraph isomorphic ****to another graph G' *= {*V'*, *E'*, ${\Sigma^{\prime }}_{V}$, ${\Sigma^{\prime }}_{E}$, *λ'*} *if there exists an injection f *: *V *→ *V' such that*

• ∏_*u*∈*V*_*M*_*λ*(*u*),*λ'*(*f*(*u*)) _≥ *τ*, *and*

• $\prod_{(u,v)\in E}{M^{\prime }}_{\lambda (u,v),\lambda^{\prime }(f(u),f(v))}\geq \tau^{\prime }$

The injection *f *is an *approximate subgraph isomorphism *between *G *and *G'*. *M *is a compatibility matrix for node label sets Σ_*V *_∪ ${\Sigma^{\prime }}_{V}$. *M' *is a compatibility matrix for edge label sets Σ_*E *_∪ ${\Sigma^{\prime }}_{E}$. In an edge compatibility matrix, we assume Σ_*E *_and ${\Sigma^{\prime }}_{E}$ both contain a special label called empty edge. In this way, we handle both topology distortion (missing edges) and edge label mismatches in the same unified way through an edge compatibility matrix. *τ *(0 <*τ *≤ 1) is the threshold for node mismatch and *τ'*(0 <*τ' *≤ 1) is the threshold for edge mismatch.

For simplicity in the following discussion, we assume that we only need to handle node label mismatches (i.e. corresponding edge relations and corresponding edge labels should exactly match each other in matching two graphs). In principle, edge label mismatch (including missing edges) can be handled in a similar way as node label mismatch. Hence our assumption does not reduce the complexity of algorithm design, but the assumption significantly simplifies our demonstration and makes our algorithm easy of access.

With the assumption, the new definition of approximate subgraph isomorphism is:

#### Definition 4

*A graph G is ****approximate subgraph isomorphic ****to another graph G', denoted by G *⊆_*a *_*G' if there exists a 1-1 injection f V*[*G*] *to V*[*G'*], *such that*

• ∏_*u*∈*V *_*M*_*λ*(*u*),*λ'*(*f*(*u*)) _≥ *τ*,

• ∀ *u*, *v *∈ *V*, (*u*, *v*) ∈ *E *⇔ (*f*(*u*), *f*(*v*)) ∈ *E', and*

• ∀ (*u*, *v*) ∈ *E*, *λ*(*u*, *v*) = *λ*(*f*(*u*), *f*(*v*))

Given a node injection *f *from graph *G *to *G'*, the co-domain of *f *is an *embedding *of *G *in *G'*. *M *is a compatibility matrix for node label sets Σ_*V *_∪ ${\Sigma^{\prime }}_{V}$. The *approximate subgraph isomorphism score *of *f*, denoted by *S*_*f*_(*G, G'*), is the product of normalized probabilities: $S_{f}(G,G^{\prime })=\prod \frac{M_{\lambda (u),\lambda^{\prime }(f(u))}}{M_{\lambda (u),\lambda (u)}}$. For the case of exception in mutation matrix, we use *MAX*(*M*_*λ*(*u*), *_) as the normalizing factor instead of *M*_*λ*(*u*),*λ*(*u*)_. For a pair of graphs, there may be many different ways of mapping nodes from one graph to another and hence may have different approximate isomorphism scores. The *approximate matching score *(score for simplicity) between two graphs, denoted by *S*(*G*, *G'*), is the largest approximate subgraph isomorphism score, or

$$S(G,G^{\prime })=\underset{f}{\max}\{S_{f}(G,G^{\prime })\}$$

Similarly, we define exact subgraph isomorphism below.

#### Definition 5

*A graph G is ****subgraph isomorphic ****to another graph G', denoted by G *⊆ *G' if there exists a 1-1 injection f from the node set V of a graph G to V' of a graph G', such that*

• ∀ *u *∈ *V*, *λ*(*u*) = *λ'*(*f*(*u*))

• ∀ *u*, *v *∈ *V*, (*u*, *v*) ∈ *E *⇔ (*f*(*u*), *f*(*v*)) ∈ *E'*, *and*

• ∀ (*u*, *v*) ∈ *E*, *λ*(*u*, *v*) = *λ*(*f*(*u*), *f*(*v*))

**Example 2. ***In *Figure 2, *we show a graph database D *= {*P*, *Q*, *R*} *and a compatibility matrix M. We set isomorphism threshold τ *= 0.4 *and with this threshold, graph P is approximate subgraph isomorphic to graph Q with the approximate subgraph isomorphic score equaling *0.6. *To see this, there are a total of 6 different ways to map nodes of P to those of Q. The only two that satisfy edge label constraints are f*_1 _= *p*_1 _→ *q*_1 _*p*_2 _→ *q*_2 _*p*_3 _→ *q*_3 _*and f*_2 _= *p*_1 _→ *q*_2 _*p*_2 _→ *q*_1 _*p*_3 _→ *q*_3_. *The approximate subgraph isomorphism score of f*_1 _*equals that of f*_2_.

#### Definition 6

*Given a graph database D, an isomorphism threshold τ, a support threshold σ *(0 <*σ *≤ 1), *the ****support value ****of a graph G, denoted by sup*_*G*_, *is the average score of the graph to graphs in the database:*

(1) $$supG=\sum_{G^{\prime }\in D,G\subseteq_{a}G^{\prime }}S(G,G^{\prime })/|D|$$

*G *is a *frequent approximate subgraph *if its support value is at least *σ*. With this definition, we only use those graphs that a subgraph *G *is approximate subgraph isomorphic to (controlled by the parameter *τ*) to compute the support value of *G*. We do this to filter out low quality (but potentially many) graph matchings in counting the support value of a subgraph. For a moderate sized graph database (100 1000), according our experience, the number of frequent subgraphs identified is usually not sensitive to the isomorphism threshold, which makes sense since low quality graph matching has low "weight" in the support computation nevertheless.

#### Problem statement

**Figure 3:**
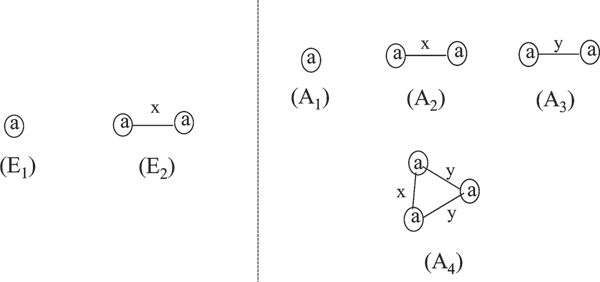
**Example of frequent subgraphs and approximate frequent subgraphs**. Given the graph database *D *in *Figure *2 and the support threshold *σ *= 2/3,the left side shows the frequent subgraphs mined by the general exact graph mining. Given the compatibility matrix *M *in *Figure *2, isomorphism threshold *τ *= 0*:*4, and support threshold *σ *= 2/3. The right side presents the frequent approximate subgraphs in *D*.

Given a graph database *D*, an isomorphism threshold *τ*, a compatibility matrix *M*, and a support threshold *σ*, the **approximate subgraph mining **problem is to find all the frequent approximate subgraphs in *D*. In Figure 3, we show all the frequent approximate subgraphs in the graph database *D *shown in Figure 2. By comparison with the frequent subgraphs acquired by the exact graph mining, the approximate mining method identifies meaningful patterns that cannot be identified by exact graph mining methods. Since the support value of approximate subgraph mining and that of frequent subgraph mining have different meaning, it is generally hard to do a comparison of approximate subgraph mining and that of frequent subgraph mining. Fortunately with the assumption of stable compatibility matrix, we can see frequent subgraph mining as a special case of approximate subgraph mining.

**Example 3. ***Given a graph database D, a compatibility matrix M in *Figure 2, *the support threshold σ *= 2/3 *and isomorphism threshold τ *= 0*:*4, *we show how to calculate the isomorphism score and support value for the approximate frequent patterns in *Figure 3.

*S*(*A*_1_, *P*) = 1, *S*(*A*_1_, *Q*) = 1, *S*(*A*_1_, *R*) = 1, *Sup*(*A*_1_) = 3/3;

*S*(*A*_2_, *P*) = 1, *S*(*A*_2_, *Q*) = 1, *S*(*A*_2_, *R*) = 1, *Sup*(*A*_2_) = 3/3;

*S*(*A*_3_, *P*) = 1, *S*(*A*_3_, *Q*) = 0.6, *S*(*A*_3_, *R*) = 0.4, *Sup*(*A*_3_) = 2/3;

*S*(*A*_4_, *P*) = 1, *S*(*A*_4_, *Q*) = 0.6, *S*(*A*_4_, *R*) = 0.4, *Sup*(*A*_4_) = 2/3.

### Algorithm design

Here we demonstrate a new algorithm APGM for approximate subgraph mining. APGM starts with frequent single node subgraphs. At a subsequent step, it adds a node to an existing pattern to create new subgraph patterns and identify their support value. If none of the resulting subgraphs are frequent, APGM backtracks. APGM stops when no more patterns need to be searched. Before we proceed to the algorithmic details, we introduce the following definitions to facilitate the demonstration of the APGM algorithm.

#### Definition 7

*Given a graph T, one of the embeddings e *= *v*_1_, *v*_2_,⋯,*v*_*k *_*of T, a node v is a ****neighbor ****of e if ∃u *∈ *e*, (*u*, *v*) ∈ *E*[*G*].

In other words, a neighbor node of a embedding *e *is any node that connects to at least one node in *e*. The *neighbor set *of an embedding *e*, denoted by *N*(*e*), is the set of *e*'s neighbors.

#### Definition 8

*Given a graph T, one of the embeddings e *= *v*_1_, *v*_2_,⋯,*v*_*k *_*of T in a graph G, a node v ∈ N*(*e*), *and a node label l, the ****approximate subgraph***, *denoted by *G|_*T*,*e*,*v*,*l*_, *is a graph *(*V', E'*, ${\Sigma^{\prime }}_{V}$, ${\Sigma^{\prime }}_{E}$, *λ'*) *such that*

• *V' *= {*v*_1_, *v*_2_,⋯,*v*_*k*_} ∪ *v*

• *E' *= *V' *× *V' *∩ *E*[*G*]

• ${\Sigma^{\prime }}_{V}$ = Σ_*V*_

• ${\Sigma^{\prime }}_{E}$ = Σ_*E*_

• ∀ *u *∈ *e *: *λ'*(*u*) = *λ*_*T*_(*u*)

• *λ'*(*v*) = *l*

• ∀ *u*, *v *∈ *e *: *λ'*((*u*, *v*)) = *λ*_*G*_((*u*, *v*))

**Figure 4:**
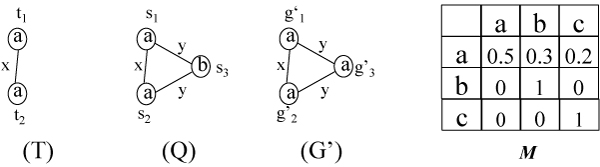
**Approximate subgraph**. A pattern *T*, a graph *Q *and approximate subgraph *G' *of *Q*.

**Example 4. ***In *Figure 4, *we show a pattern T and one of its embeddings e *= (*s*_1_, *s*_2_) *in a graph Q. Node s*_3 _*is a *neighbor node *of e since it connects to at least one node of e (in fact both). Given a node label l ="a", we obtain an approximate subgraph G' *= *Q*|_*T*,*e*,*v*,*l*_*of Q shown in the same figure. The G' has an embedding e' *= (*s*_1_, *s*_2_, *s*_3_) *in Q and the score of the embedding is *$\frac{M(a,a)}{M(a,a)}\frac{M(a,a)}{M(a,a)}\frac{M(a,b)}{M(a,a)}=\frac{M(a,b)}{M(a,a)}=0.6$. *(Recall the score of an embedding is the multiplication of the probability of observed node label replacement, normalized by the probability of node label self-replacement.)*

With the two definitions, we present the pseudo code of APGM below. follows.

**Algorithm 1. ***APGM_MAIN(D, M, τ, σ)*

1: Begin

*2: C *← {*frequent single node*}

*3: F *← *C*

*4: **for **each T *∈ *C ****do***

*5:    APGM_SEARCH*(*T*, *τ*, *σ*, *F*)

*6: ****end for***

7: return F

8: End

**Algorithm 2. ***APGM_SEARCH(T, τ, σ, F)*

1: Begin

*2: C *← ∅

*3: **for **each *(*e, v*), *e is an embedding of T in G, v *∈ *N*(*e*) ***do***

*4:    CL *← *approximateLabelSet(T, G, e, v)*

*5:    **for **each l ∈ CL ****do***

*6:       X *← *G*|_*T*, *e*, *v*, *l*_

*7:       C *← *C *∪ {*X*}

*8: *      H(*X*) = H(*X*) ∪ (*e*, *v*)

*9: ***   *end for***

*10: ****end for***

11: remove infrequent T from C

*12: F *← *F *∪ *C*

*13: **for **each T *∈ *C ****do***

*14:    APGM_SEARCH*(*T*, *τ*, *σ*, *F*)

*15: ****end for***

16: End

H is a hash function to store candidate subgraphs and their embeddings. The hash key of the function in our implementation is a canonical code of the subgraph *X*, which is a unique string presentation of a graph. We use the Canonical Adjacency matrix (CAM) and the Canonical Adjacency Matrix code, developed in [48], to compute the canonical code of a graph.

**Algorithm 3. ***approximateLabelSet(T, G, e, v)*

1: Begin

*2: R *← ∅

*3: l*_0 _← *λ*_*G*_(*v*)

*4: **for **each l *∈ Σ_*V*[*G*] _***do***

*5: ***   *if ***$S(e,T)\times \frac{M(l_{0},l)}{M(l_{0},l_{0})}\geq \tau$ ***then***

*6:       R *← *R *∪ *l*

*7: ***   *end if***

*8: ****end for***

9: return R

10: End

**Example 5. ***Applying APGM to the graph database shown in *Figure 2*with the support threshold σ *= 2/3 *and the isomorphism threshold τ *= 0.4, *we identify one frequent single-node pattern a (shown as A*_1 _*in *Figure 3*). Adding one node to the pattern A*_1_, *there are two candidate single-edge patterns and both of them are frequent. These two are shown as A*_2 _*and A*_3 _*in the same figure. From pattern A*_2_, *we enumerate one additional pattern A*_4_. *We stop here since there is no more candidate patterns to explore*.

## Results

### Experimental setup

We performed all the experiments on a cluster with 256 Intel Xeon 3.2 Ghz EM64T processors with 4 GB memory each. The approximate graph mining algorithm was implemented in the C++ language and compiled by using the g++ compiler in Linux environment with -O3 optimization.

We downloaded all protein structures from Protein Data Bank (PDB). We followed [45] to use the same software as [47] to calculate Almost-Delaunay(AD) for graph representation of protein geometry. We took BLOSUM62 as the compatibility matrix and back-calculated the conditional probability matrix by following the procedure described in [49]. We normalized the matrix according to *Definition*4.

### Data set

We investigated two immunologically relevant protein domain families: the Immunoglobulin V set and the Immunoglobulin C1 set. Immunoglobulin domains are among those used by immunoevasins [50,51]. We collected proteins from SCOP release 1.69. For each family we created a culled set of proteins with maximal pairwise sequence identity percentage below some threshold by using PISCES server [52](Immunoglobulin C1 set below 40%, and Immunoglobulin V set below 30%). The characteristics of the complete domain sequence sets are shown in Table 1. And the PDB IDs of individual proteins for the two culled sets are shown in Table 2.

**Table 1:** Characteristics of domain sequence sets

	Immunoglobulin C1 Set	Immunoglobulin V Set
Number of Proteins	1786	371

Average Length	210	194

Maximum Length	457	444

Minimum Length	98	99

**Table 2:** Immunoevasins protein lists for research

	PDB ID of proteins in Immunoglobulin C1 set
Proteins for Feature Extraction(10):	1*fp*5*a *1*onqa *1*ogad *1*pqza *1*t*7*va *1*l*6*xa *1*je*6*a *1*mjul *1*uvqb *1*dn*0*b*

Proteins for Leave-one-out Testing(11):	1*nfda *1*uvqa *1*q*0*xl *1*mjuh *1*a*6*za *1*k*5*na *1*hdma *3*frua *1*ogae *1*hdmb *1*k*5*nb*

	PDB ID of proteins in Immunoglobulin V set

Proteins for Feature Extraction(10):	1*pkoa *1*ogad *1*npua *1*cdca *1*jmaa *1*fo*0*b *1*nkoa *1*mjuh *1*nfdb *1*qfoa*

Proteins for Leave-one-out Testing(9):	1*zcza *1*f*97*a *1*eaja *1*mjul *1*cida *1*neua *1*cdya *1*hkfa *1*nezg*

### Experimental protocol

**Figure 5:**
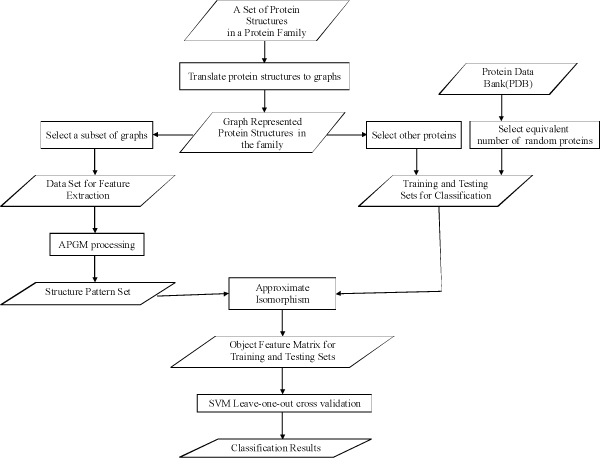
The procedure of experimental research.

We randomly divided proteins from each family into two groups: 10 proteins to serve as sources for feature extraction, and the remainder(positive sample) for training and testing in "leave-one-out" cross validation. A negative sample set of the the same size as the positive sample set was randomly chosen from PDB. The negative sample was used along with the positive sample in testing. The complete flowchart of our experiment procedure is shown in Figure 5. During this experimental research, we mined frequent clique subgraphs [53] in order to enforce biological constraints on the patterns. We compared APGM with the exact graph mining methods MGM [53]. We chose MGM as the counterpart for the comparison because it is an available clique pattern mining algorithm. (Any exact match method with clique constraint should provide the same number of patterns from a graph database.)

### Number of patterns identified

**Figure 6:**
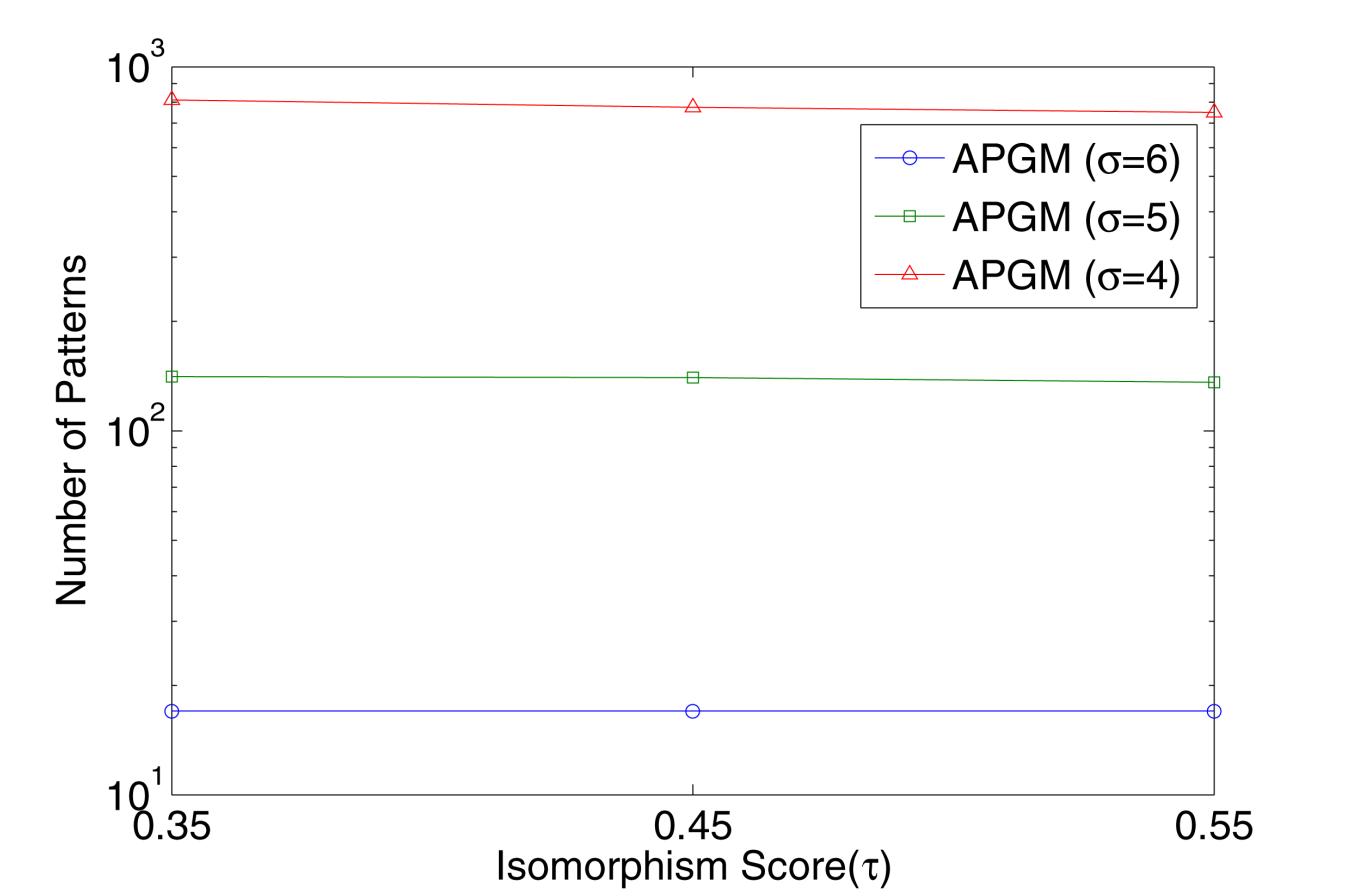
**Number of patterns for Immunoglobulin C1 set acquired by APGM**. Example of a graph database *D *and a compatibility matrix *M*.

We identified frequent approximate subgraph patterns from 10 positive proteins in each family. There are two parameters that may have significant influence on the set of mined patterns. The first is the support threshold(*σ*) and the second is the isomorphism threshold(*τ*). For simplicity, in following experiments in this section we use the new support threshold *σ' *= *σ *× |*D*|, |*D*| is the size of graph database, and the same change applied in support value. In Figure 6, we run APGM with different combinations of *τ *and *σ *and collect the total number of identified patterns. Our results show that the total number of patterns is not sensitive to the isomorphism threshold, and rather depends on the support threshold heavily. Such fact eases the worry that the parameter *τ *may be too strong for deciding the number of patterns.

For the purpose of comparison, the number of patterns mined by two mining methods are shown in Table 3 and 4, and the number of patterns acquired by APGM from Immunoglobulin C1 proteins are also shown in Figure 6. In our experiment, we treat a pattern set with the number more than 10000 as a meaningless one because our sample space is comparatively small and the isomorphism check is computationally expensive. From Table 4, we see that exact match fails to provide useful patterns on the Immunoglobulin V proteins, which is the typical data set with very noisy background. In comparison, APGM does find some pattern set with a reasonable size in such situation. (We only use rough parameter combination grids to do the pattern search. If we increase the precision of *τ *and *σ*, more patterns will be found.) In order to evaluate the quality of these patterns, we use the identified frequent subgraphs in classification tests as discussed below.

**Table 3:** Number of patterns by APGM(*τ *= 0.35) and MGM on Immunoglobulin C1

	Support Threshold(*σ*)				
	
	6	5.5	5	4.5	4
*APGM*(*τ *= 0.35)	17	24	141	202	841

*MGM*	16	16	126	126	660

**Table 4:** Number of patterns by APGM(*τ *= 0.75) and MGM on Immunoglobulin V

	Support Threshold(*σ*)				
	
	6	5.5	5	4.5	4
*APGM*(*τ *= 0.75)	0	0	0	160	14686

*MGM*	0	0	0	0	13911

### Classification performance

In this experimental section, we used *libsvm *SVM package [54] for protein structure classification. We treat each mined pattern as a feature and a protein is represented as a feature vector *V *= (*v*_*i*_) where *i *≤ *i *≤ *n *and *n *is the total number of identified features. *v*_*i *_is 1, if the related feature occurs in the protein and otherwise *v*_*i *_is 0. We used the linear kernel and default parameters for SVM leave-one-out cross validation. The classification results are summarized in Table 5 and 6. For some parameter combinations, there are no accuracies – an event which happens under two circumstances. First, there are no patterns found. Second, the pattern set is too big to be useful. From the tables we see that the classifications with APGM-based feature highly outperform those based on exact match. For Immunoglobulin C1 set, the classification based on feature identified by MGM only can reach 73%, while APGM is between 69%~91%. For Immunoglobulin V set, since the exact match method cannot mine any meaningful patterns, it fails in classification, while by using APGM, we have the accuracy around 78%. This shows that our APGM has more capability to mine useful structure information from very noisy background than general exact match graph mining algorithms.

**Table 5:** Classification accuracy of APGM (*τ *= 0.35) and MGM on Immunoglobulin C1 Set

	Support Threshold(*σ*)				
	
	6	5.5	5	4.5	4
*APGM*	68.18%	77.27%	86.36%	90.91%	81.82%

*MGM*	72.73%	72.73%	72.73%	72.73%	72.73%

**Table 6:** Classification accuracy of APGM *τ *= 0.75) and MGM on Immunoglobulin V set

	Support Threshold (*σ*)			
	
	6	5.5	5	4.5
*APGM*	-	-	-	77.78%

*MGM*	-	-	-	-

TP, true positive; FP, false positive; TN, true negative; FN, false negative.

Accuracy = (TN+TP)/(TN+TP+FN+FP).

- means accuracies are unavailable.

### Statistical significance of patterns

**Figure 7:**
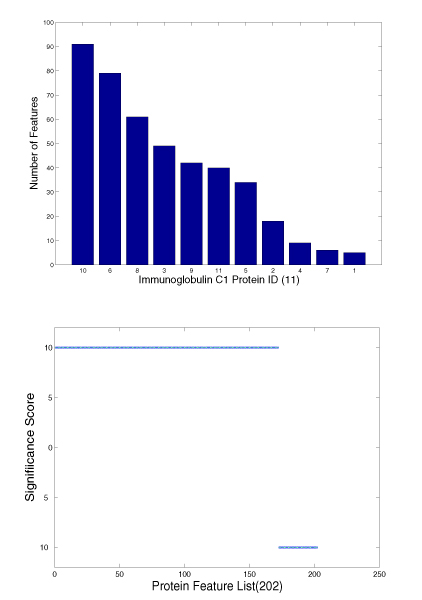
**Distribution and significance of features among Immunoglobulin C1 Proteins. Upper: **Distribution of frequent subgraph features among Immunoglobulin C1 proteins. **Lower: **Significance of frequent subgraph features among Immunoglobulin C1 proteins. Both figures are constructed for the set for classification. There are 202 patterns that are mined with the support threshold *σ *= 4.5 and the isomorphism threshold *τ *= 0.35.

**Figure 8:**
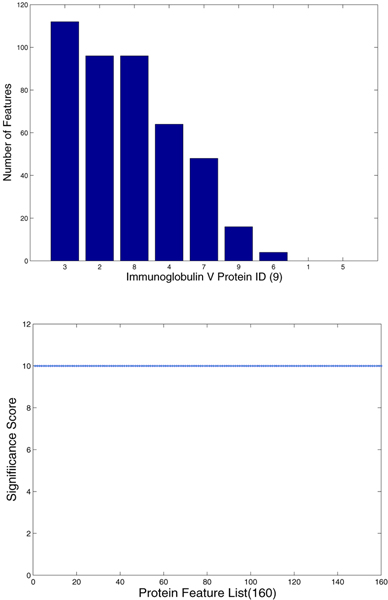
**Distribution and significance of features among Immunoglobulin V proteins. Upper: **Distribution of frequent subgraph features among Immunoglobulin V proteins. **Lower: **Significance of frequent subgraph features among Immunoglobulin V proteins. Both figures are constructed for the set for classification. There are 160 patterns that are mined with the support threshold *σ *= 4.5 and the isomorphism threshold *τ *= 0.75.

In order to further demonstrate the quality of the patterns mined by using APGM, we chose the parameter combination with the best accuracy for the Immunoglobulin C1 proteins and the Immunoglobulin V proteins to check the distribution and significance of patterns. Figure 7 shows the number of the patterns that the 11 Immunoglobulin C1 proteins contain and the significance scores. Figure 8 shows those for the 9 Immunoglobulin V proteins. Proteins in Figure 7 and 8 are numbered according to their appearance order in in Table 2. For example protein "10" in Figure 7 is protein 1nfa (chain A). The proteins in Figure 7 and 8 are sorted according to the number of patterns contained in the proteins. The significance score *P *is defined as follows.

(2) $$\begin{array}{cc} P=\log\frac{f^{+}/N^{+}}{f^{-}/N^{-}},if\;f^{-}\neq 0 & f^{+}\neq 0 \end{array}$$

There are three special cases of *P*'s value. If *f*^- ^= 0 and *f*^+ ^≠ 0, we set *P *= 10; if *f*^- ^= 0 and *f*^+ ^≠ 0, we set *P *= -10; and if *f*^- ^= 0 and *f*^+ ^= 0, we set *P *= 0.

Although the patterns do not distribute uniformly among Immunoglobulin C1 proteins, they cover all the positive proteins. The significance score of these patterns shows strong bias toward the Immunoglobulin C1 proteins, and among 202 only 30 noise features(*P *= -10) exist. For Immunoglobulin V proteins, the features miss two positive proteins, but these features are highly correlated with positive samples with all *P *equalling 10.

### Computational performance

Since the support value of approximate subgraph mining and that of frequent subgraph mining have different meaning, it is generally hard to compare the computational performance of approximate subgraph mining and that of frequent subgraph mining. If *τ *is less than 1, approximate subgraph mining may obtain more patterns than that of general frequent subgraph mining by taking more running time. Because of this reason, we use the *pattern discovery rate *("rate" for simplicity), which is computed as the number of discovered patterns *N *divided by the running time *t*. We use rate rather than running time as the criteria to compare computational efficiencies of different algorithms. We evaluated the computational efficiency of APGM with synthetic data sets.

We generated the synthetic data set by the same synthetic graph generator as [56]. The synthetic graph generator takes the following set of parameters: *D *is the total number of graphs; *T *is the average size of graph; *I *is the average size of potentially frequent subgraphs; *L *is the number of potentially frequent subgraphs; *V *is the number of vertex labels; *E *is the number of edge labels. The default parameter values that we use are *D *= 10000, *T *= 30, *I *= 11, *L *= 200, *E *= 20, *V *= 20.

**Figure 9:**
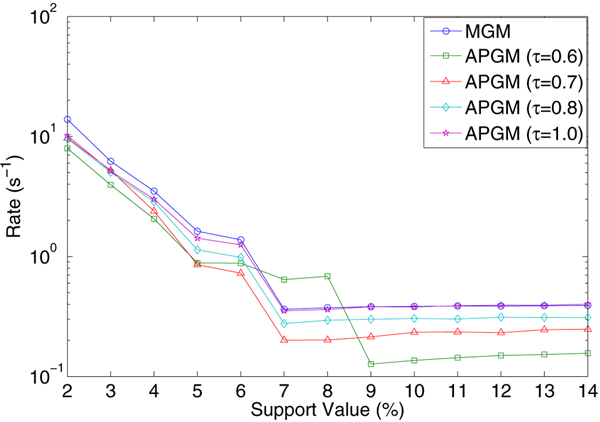
**Computational performance comparison**. We compared the computational performance between APGM and MGM using synthetic data sets. APGM used isomorphism threshold *τ *= 1.0, 0.8, 0.7, 0.6. Given the patterns' number *N *and running time *t *(*s*), *rate *= *N/t*.

We compare the performance rate between MGM and APGM using different isomorphism threshold values (and hence introduce different level of approximate matching). We use the support threshold (*σ*) defined in *Definition*6 in this experiment. From Figure 9, we see that with the change of isomorphism threshold, performance of APGM differs narrowly. Even if APGM takes approximate matching, its performance is very similar with MGM. Indeed, with some values of support threshold, APGM with low isomorphism threshold (*τ *= 0.6) even has much higher rates.

## Discussion

Finding features (corresponding to packing motifs) that discriminate one protein family from random selected proteins motivated us to further investigate the possibility of examining these motifs as characteristic signatures of a protein family. We investigated the spatial distribution of the residues covered by our mined structure motifs in individual proteins. We found the residues of structure motifs are highly centralized on a limited number of positions for each protein. We picked up the protein 1mju (chain l) in Immunoglobulin C1 set as one example. 202 patterns, which we obtained, maps to 21 amino acids among the total of 219 residues in 1mju. Through literature search, we found residues identified by APGM are related to the known functional sites in the protein. For example, position 200 and 202 are residues in contact with ligand GOL1406 as studied in [55]. Both positions are not discovered by the exact pattern mining method. This result suggests that APGM is more sensitive in recognizing functional related residues, as compared to exact pattern mining methods. However, we admit that comprehensive experimental study, involving multiple protein families, is needed before we could draw the conclusion convincingly.

## Conclusion

In this paper we present a novel data mining algorithm, **APGM**(**AP**proximate **G**raph **M**ining), to perform structure comparison and structure motif identification in diverse proteins. In our method we encode structural motifs as subgraphs of geometric graph of proteins. Instead of using a general graph mining method to extract frequent subgraph motifs, we have developed the approximate graph mining algorithm and taken advantage of known substitution matrices in protein structure motif identification. Compared with general graph mining algorithms, APGM not only offers more qualified patterns that achieve higher classification accuracy, but also shows a reasonable computational performance. By applying this method to other protein families, "structure fingerprints" can be collected and used in domain classification schemes where structural information is desired. Furthermore, without loss of generality, choice of appropriate compatibility matrices allows our method to be employed in any domain where subgraph labels have some uncertainty. For example, networks of personal contacts "mutate" as people die or change employment. Compatibility matrices assigning probabilities of 'label substitution' within families or organizations may allow the essential natures of personal contact subgraphs to be preserved nevertheless.

## Competing interests

The authors declare that they have no competing interests.

## Authors' contributions

YJ developed methods, implemented the software, and drafted the manuscript. VB and JZ were involved in testing the data set. JH was responsible for all aspects of the project, and helped revise the manuscript. LC provided advices on the biological aspect of the work, and helped revise the manuscript.
